# Ontario County Medical Society

**Published:** 1858-11

**Authors:** 


*Ontario County Medical Society.*—The annual meeting of the Ontario County Medical Society, was held at the Court House, July 13th, and was represented by twenty-five members. Prof. Maxson, president of the society, called the meeting to order at 10½ o'clock, A. M., when the proceedings of the last regular meeting were read and approved, after which several members presented some very interesting medical cases, which were discussed and commented upon, after which the annual address of the president was delivered by him. *Subject:* "Bilious Pneumonia." The address was an able production, and displayed sound and discreet knowledge of the subject treated upon.

A vote of thanks was voted to the President as a token of respect for his entertaining essay.

After which several members presented essays on various medical subjects, displaying that intuitive knowledge of practice essential to the successful practitioner; (after which several important regulations were adopted.)

On invitation of Dr. Geo. Cook, of Brigham Hall, Canandaigua, that the society visit the Insane Asylum at such time as would be convenient for them, 4 o'clock. P. M., was appointed, at which time the society adjourned, after electing the following officers:

*President*, Dr. E. CARR.

*Vice President*, Dr. H. A. Potter.

*Secretary*, Dr. J. R. Pratt.

*Censors*, Drs. Maxson, Cheney, Smith, Jewett and Wilber.

*Trustees of Library*, Drs. Cheney, Carr, Smith, Pratt and Jewett.

*Delegate to the State Medical Society*, Dr. H. A. Potter, of Geneva.

After visiting Brigham Hall, the society convened in the library room, and on motion, Dr. H. A. Potter was called to the chair, and Dr. J. T. Smith, appointed secretary.

On motion a committee was appointed to draft resolutions expressive of the thanks of the society. The following resolution was presented and adopted:

*Resolved,* That the Ontario County Medical Society return to Dr. Geo. Cook, their thanks for his polite invitation to visit Brigham Hall, the institution under his care, and that it hereby expresses its entire reliance in his capacity as physician to the insane, and an emphatic approval of his arrangements for their treatment and cure, as well as their admiration of the beauty of the locality selected, and the commodiousness and adaptedness of the building for the purpose for which it is used.

E. Carr, M. D., President.

H. A. Potter, M. D., Chairman.

J. T. Smith, Secretary.—*Ont. Republican Times.*

